# Supplementary figures and images for: Optical O2 Sensors Also Respond to Redox Active Molecules Commonly Secreted by Bacteria
Source: mBio. 2022 Oct 31;13(6):e02076-22. doi: 10.1128/mbio.02076-22 (PMC9765510; doi:10.1128/mbio.02076-22)

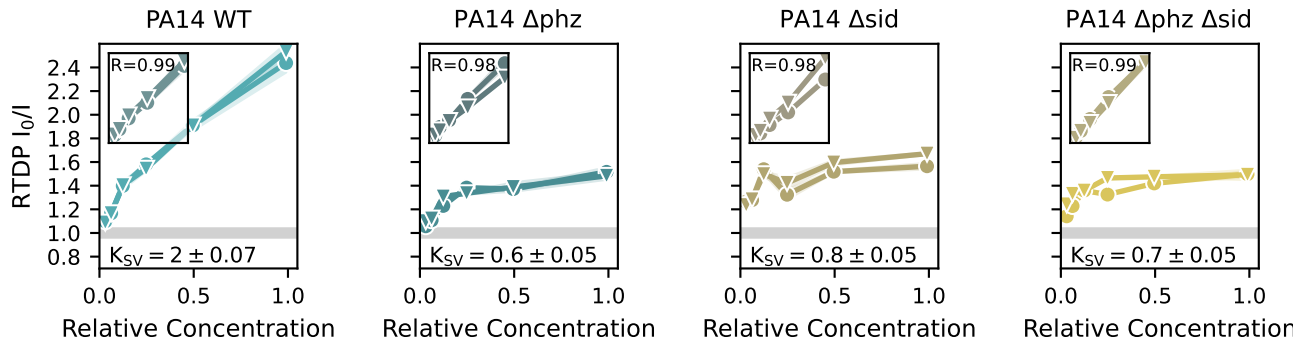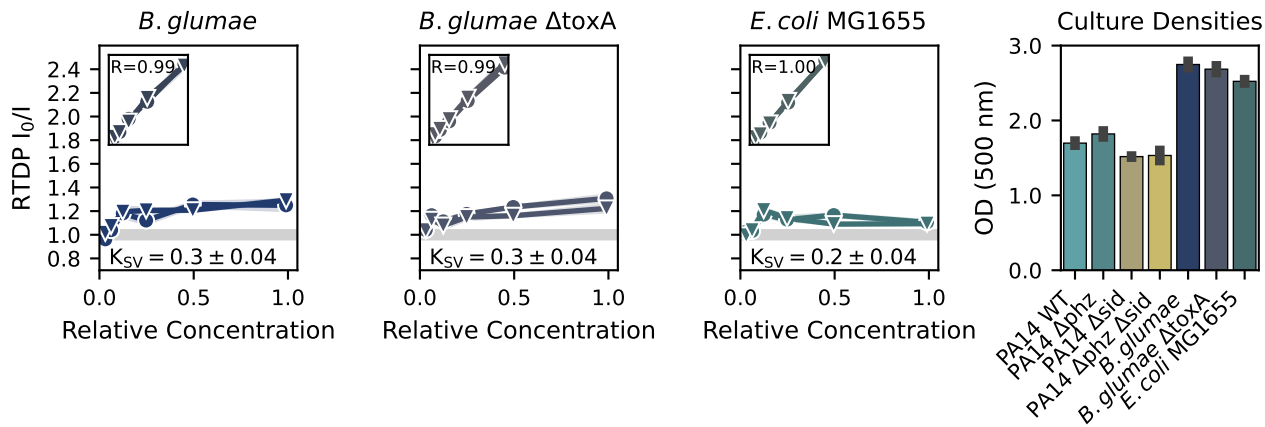

Supplement: FIG S2 [file mbio.02076-22-s0007.pdf]

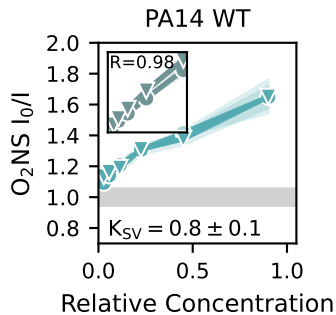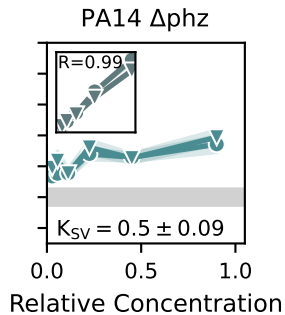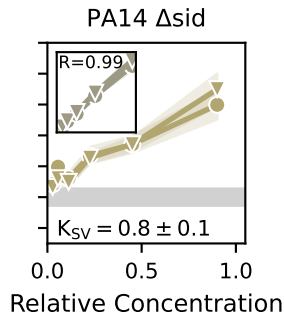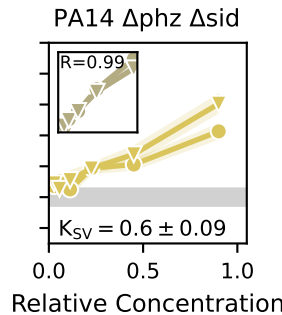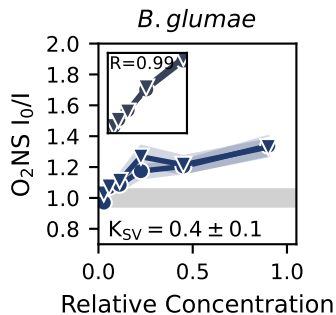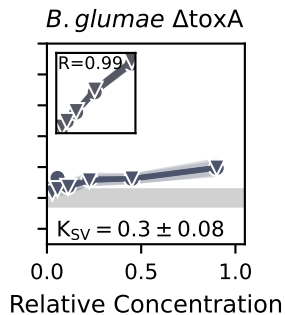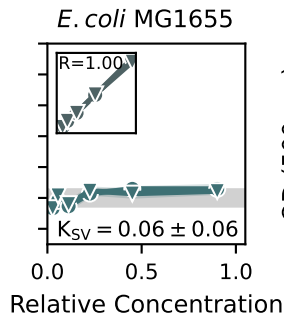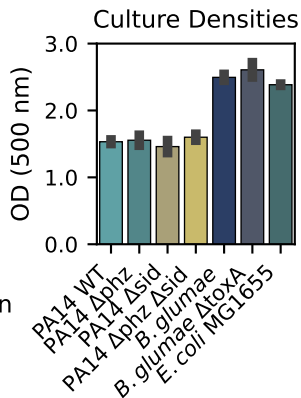

Supplement: FIG S3 [file mbio.02076-22-s0006.pdf]

**A**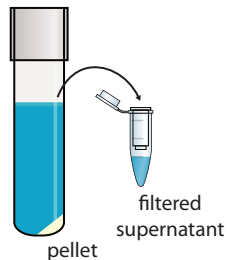**B**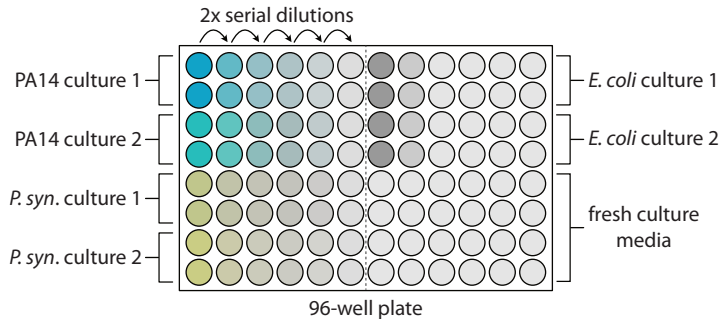**C**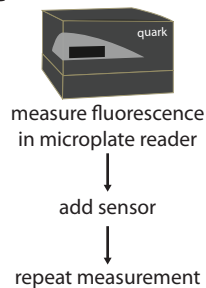**D**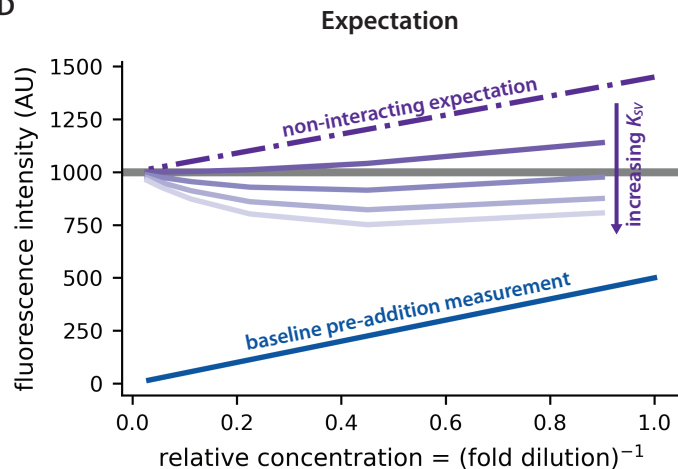**E**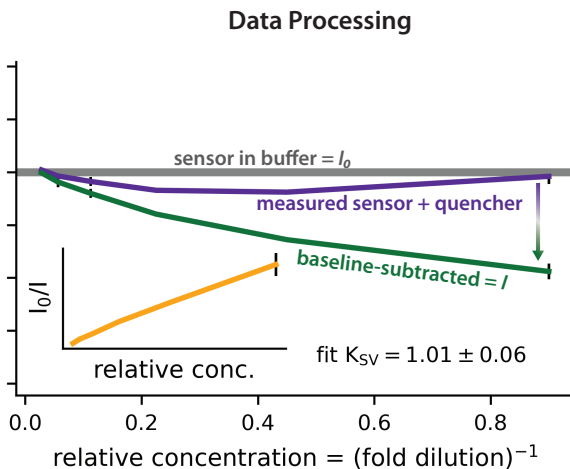

Supplement: FIG S1 [file mbio.02076-22-s0008.pdf]

Supernatants + RTDP

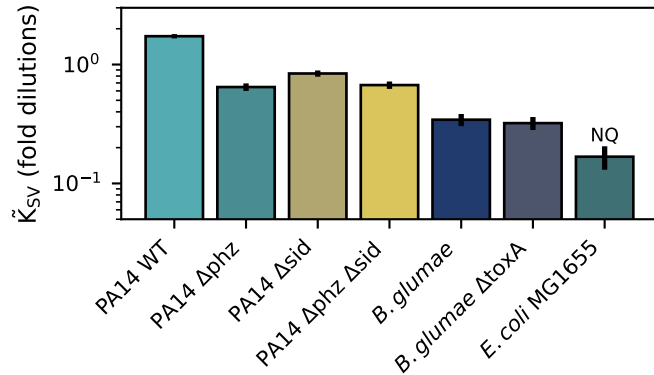Supernatants + O<sub>2</sub>NS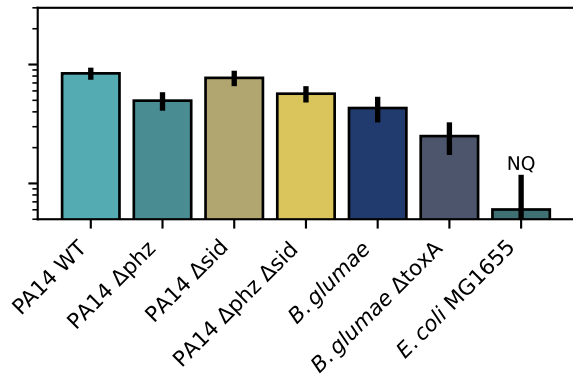

Supplement: FIG S4 [file mbio.02076-22-s0005.pdf]

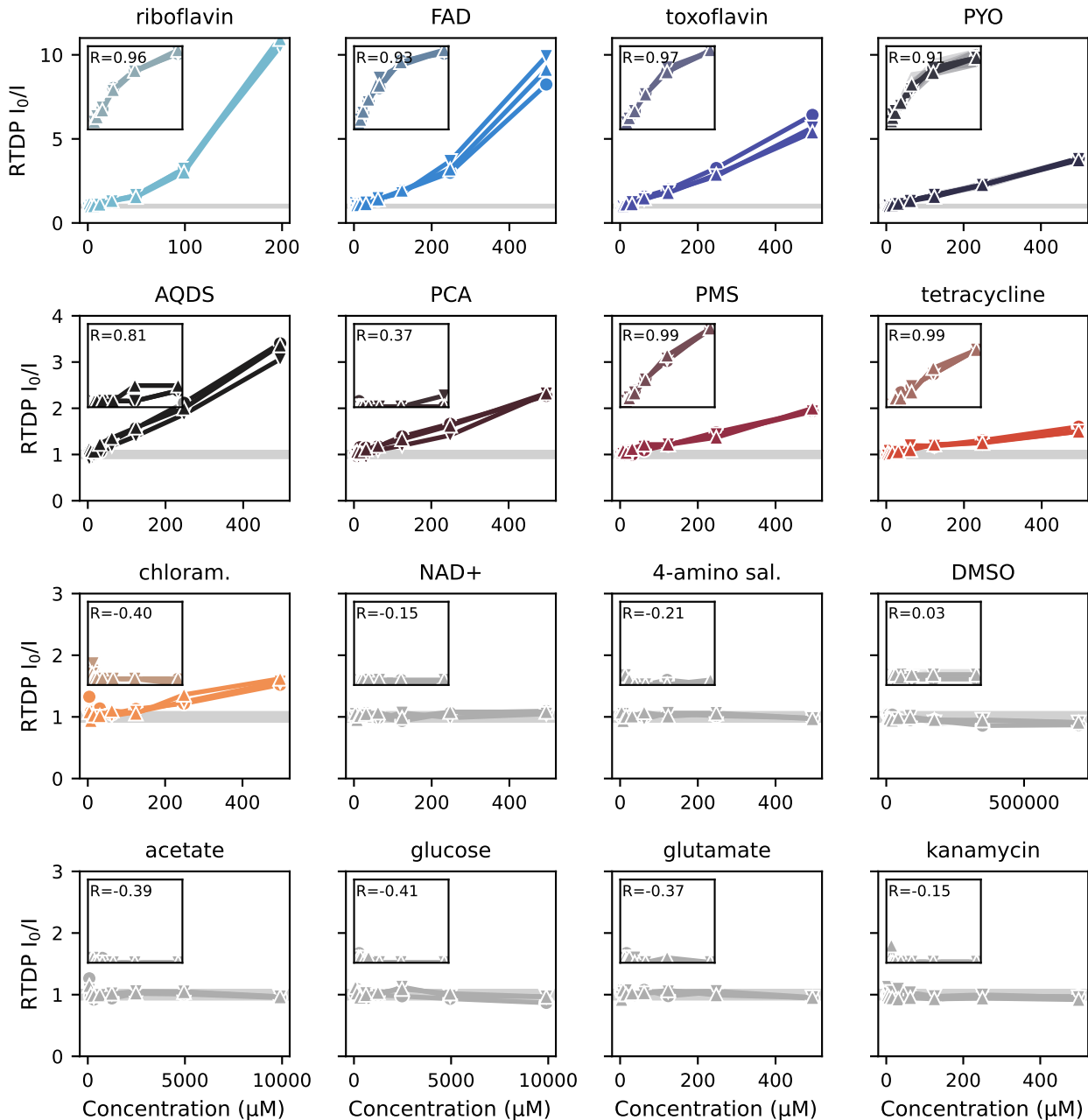

Supplement: FIG S5 [file mbio.02076-22-s0004.pdf]

toxoflavin

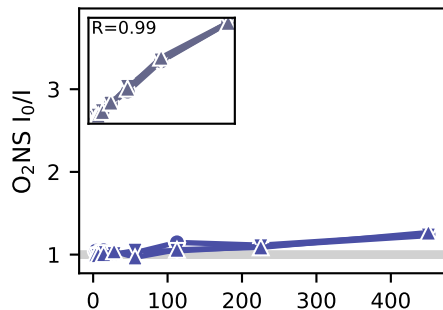

PYO

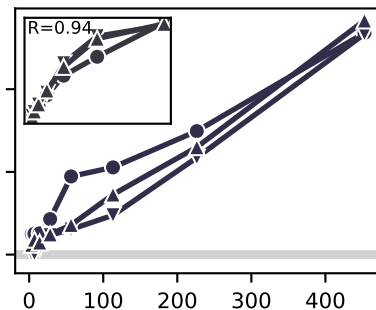

AQDS

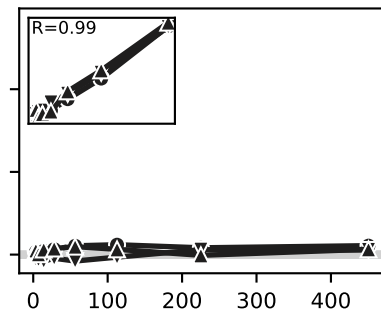

PCA

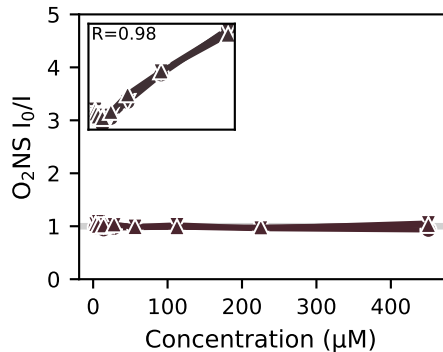

PMS

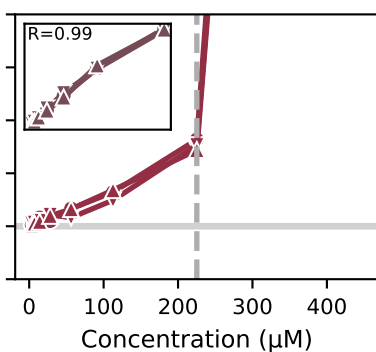

DMSO

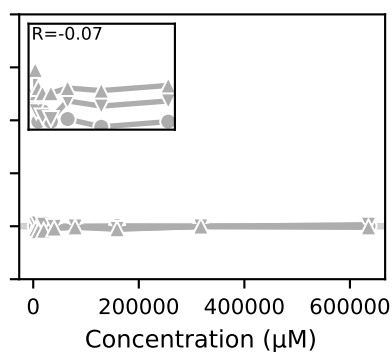

Supplement: FIG S6 [file mbio.02076-22-s0003.pdf]

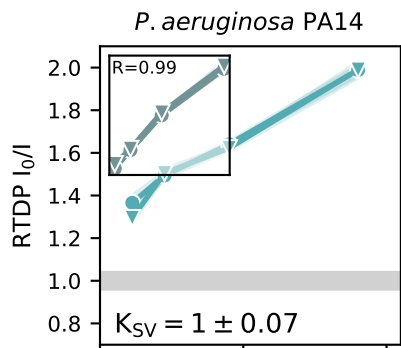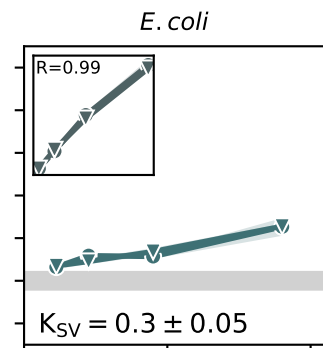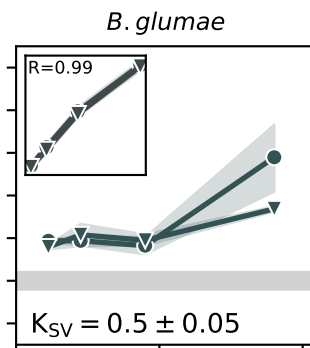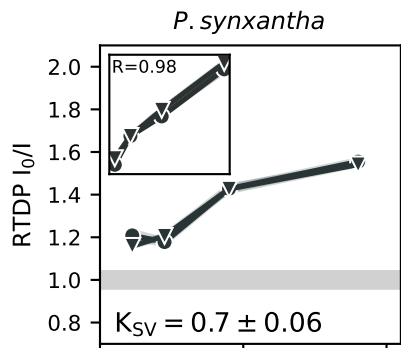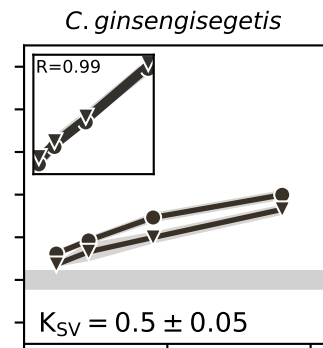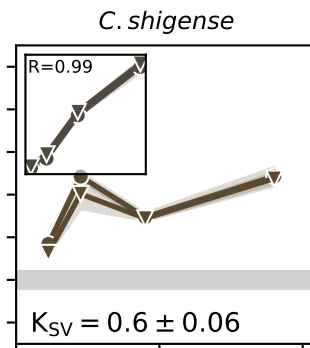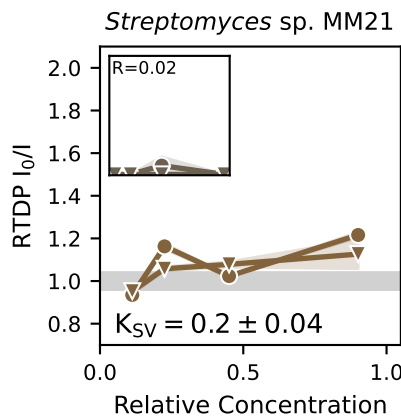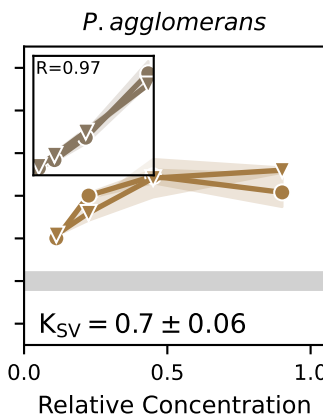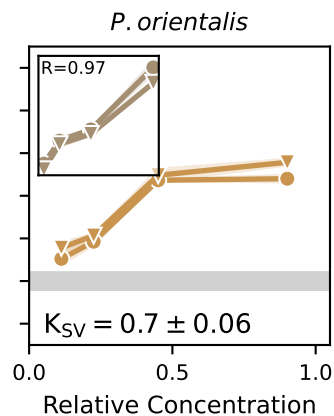

Supplement: FIG S7 [file mbio.02076-22-s0002.pdf]

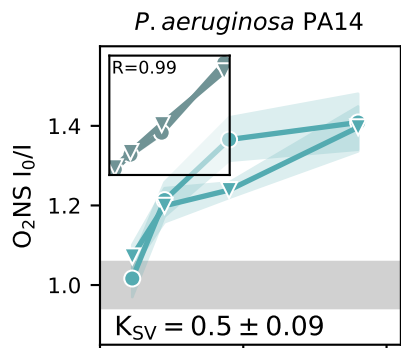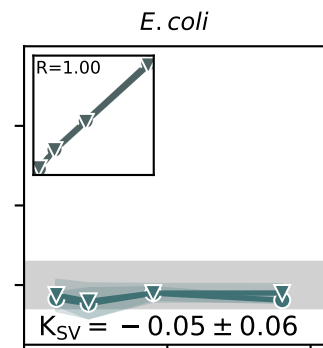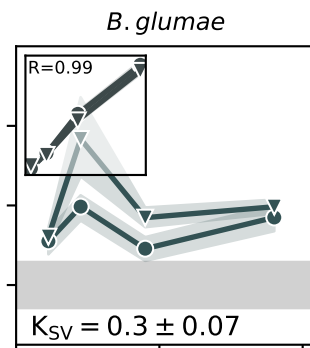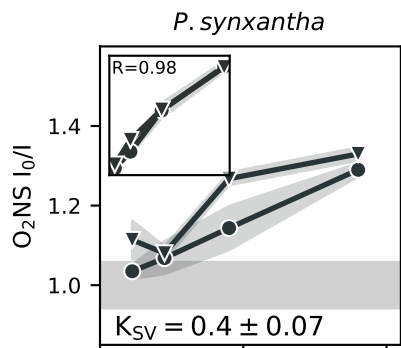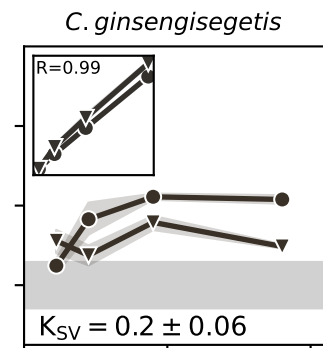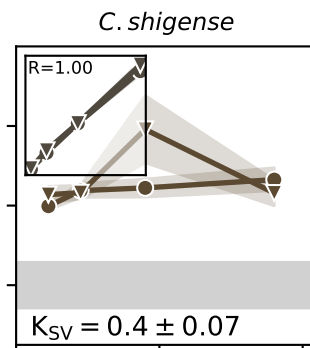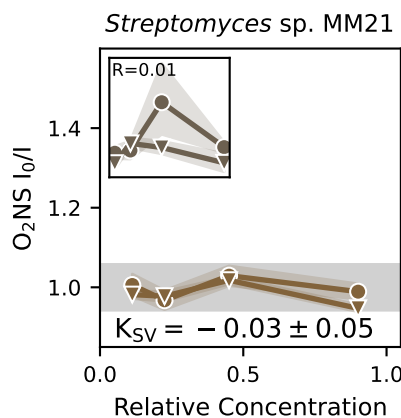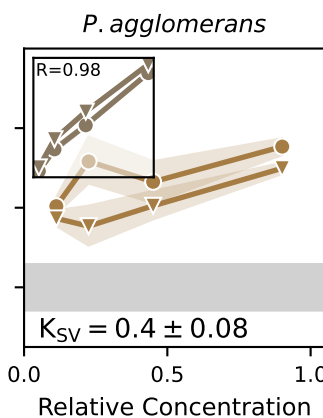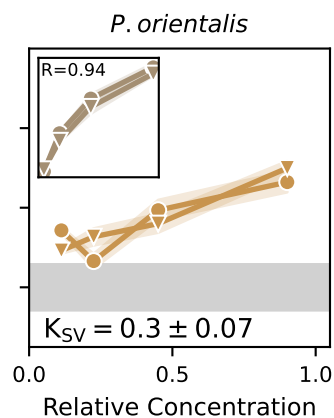

Supplement: FIG S8 [file mbio.02076-22-s0001.pdf]
